# The atypical soluble guanylyl cyclase subunit Gyc89Db does not control neuroepithelial proliferation in *Drosophila* larval brain

**DOI:** 10.17912/micropub.biology.001336

**Published:** 2024-11-01

**Authors:** Mariel Rosas, Rafael Cantera, Daniel Prieto

**Affiliations:** 1 Departamento de Biología del Neurodesarrollo, Instituto de Investigaciones Biológicas Clemente Estable, Montevideo, Uruguay; 2 Departamento de Neurofisiología Celular y Molecular, Instituto de Investigaciones Biológicas Clemente Estable, Montevideo, Uruguay

## Abstract

We investigated the role of oxygen-sensing atypical guanylyl cyclase subunit Gyc89Db in the developing brain. Despite its expression in the hypoxic neuroepithelium of the larval optic lobe of *Drosophila* , loss-of-function mutants and ectopic expression did not alter neuroepithelial cell number or proliferation. Notably, while ectopic expression of *Gyc89Db* increases optic lobe volume and neuroblast numbers, our negative results suggest that these effects manifest earlier in development without persistent alteration of the neuroepithelium, through mechanisms that might be independent of neuroepithelial proliferation.

**Figure 1. Assessment of Neuroepithelial Cell Number and Proliferation in Larval Brain.**
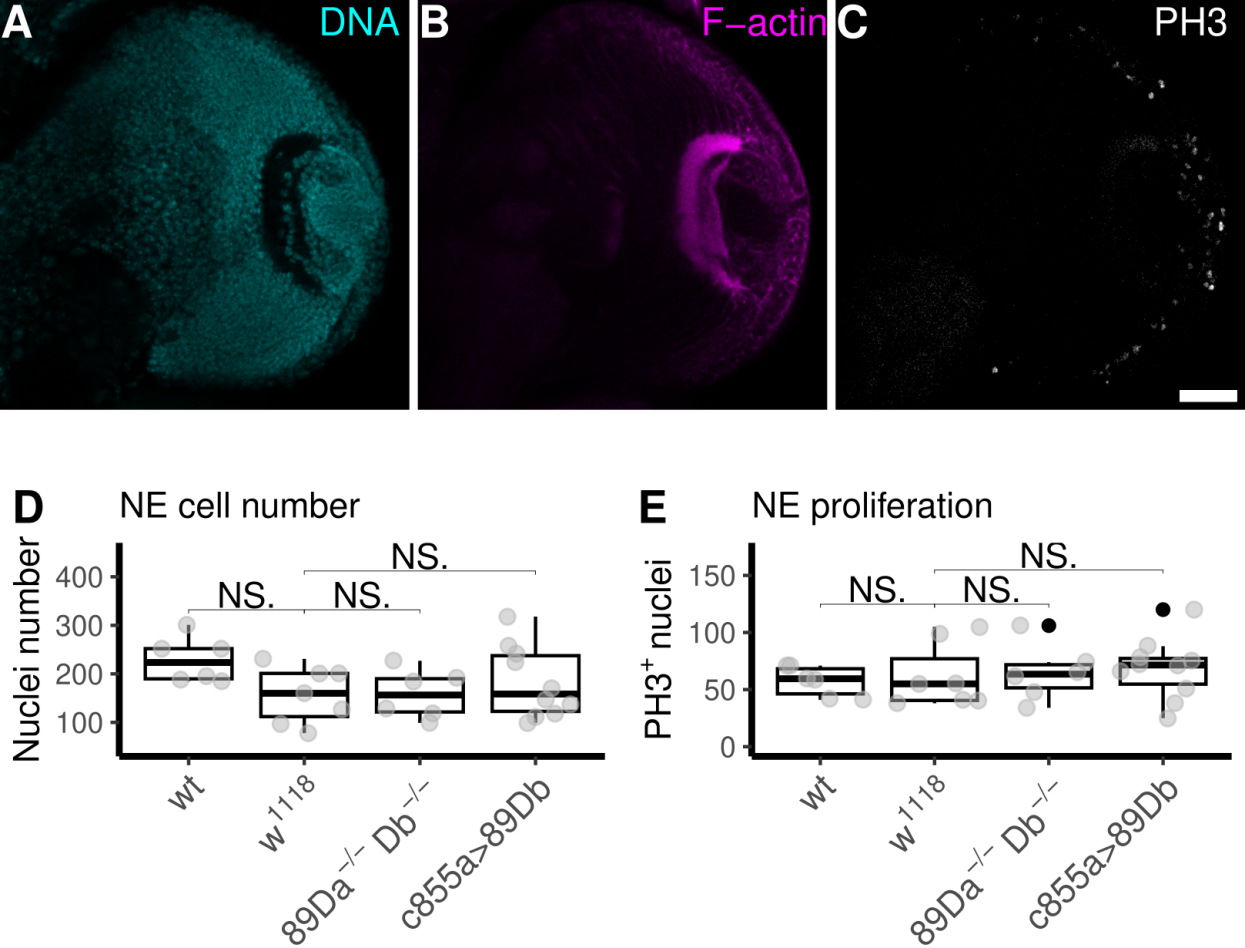
**(A-C)** show the three fluorescent markers at a single confocal optical section across a brain hemisphere of a wild-type (wt) L3 larva. **(A)** DNA staining (cyan) with methyl green shows all nuclei. **(B)** F-actin staining with phalloidin (magenta) used for the anatomical segmentation of the neuroepithelium. **(C)** Immunostaining against phosphorylated histone H3 (gray) shows mitotic nuclei for determination of the number of proliferating neuroepithelial cells. **(D)** The numbers of neuroepithelial cells found in double mutant larvae ( *89Da ^-/-^ Db ^-/-^* ; median±SD: 156.50 ± 49.87) or in larvae ectopically expressing *Gyc89Db* in the neuroepithelium ( *c855a>89Db* ; median±SD: 158.50 ± 73.83) did not differ from the numbers found in the two control genotypes ( *wt* ; median±SD: 223.5 ± 46.94 and *w ^1118^* ; median±SD: 160.00 ± 57.87). **(E)** Proliferating neuroepithelial cells were counted as cell nuclei stained positively for anti-PH3 (MG ^+^ /PH3 ^+^ ) within the segmented neuroepithelial volume. The numbers of proliferating cells in larvae carrying the double mutation *Gyc89Da ^-/-^ Db ^-/-^* ( *89Da ^-/-^ Db ^-/-^* ) (median±SD: 63.50 ± 24.58) were not different from the numbers found in larvae expressing *Gyc89Db* ectopically in the neuroepithelium ( *c855a> Gyc89Db* ) (median±SD: 71.50 ± 26.50) or in larvae from any of the two control strains *wt* (median±SD: 59.50 ± 13.31) and *w ^1118^* (median±SD: 55.00 ± 28.33) and. **N=6; Mann-Whitney U-test.** NE:=Neuroepithelium. Scale bar: 30 μm.

## Description

The neuroepithelium (NE) of the optic lobe (OL) is the major stem cell niche in the larval brain of *Drosophila melanogaster* (Hofbauer and Campos-Ortega 1990). It comprises hundreds of cells arising through symmetric proliferative divisions, progressively differentiating into self-renewing neuroblasts. These neuroblasts proliferate through asymmetric cell divisions, generating ganglion mother cells, which, in turn, give rise to glia and neurons (Egger et al. 2007; Yasugi et al. 2008) . The NE is hypoxic relative to the rest of the brain (Baccino-Calace et al. 2020) and since we were not able to observe some of the signs of a typical hypoxia response (Misra et al. 2017; Baccino-Calace et al. 2020) we wondered whether NE cells were able to sense hypoxia through some alternative way.

Three oxygen-sensing atypical guanylyl cyclases (asGCs) activated by hypoxia have been described in *Drosophila* , namely Gyc88E, Gyc89Da, and Gyc89Db (Vermehren-Schmaedick et al. 2010; Morton 2011) . These asGCs, despite being mostly inactive individually, show enhanced activity when co-expressed, suggesting functional relevance as heterodimers, particularly in the NE (Morton et al. 2005; Morton and Vermehren 2007) .

Additionally, it is accepted that the *Drosophila* asGCs exist mostly as heterodimers with some functional redundancy of the heterodimers compared to the homodimers, but the fact that *in vivo* *Gyc89Da* and *Gyc89Db* are frequently co-expressed with *Gyc88E* (Morton et al. 2005) support the idea that heterodimers might be the functionally relevant conformation.

Our previous findings demonstrated that ectopic expression of Gyc89Db in the NE increased the number of neuroblasts and OL volume (Prieto et al. 2024) . Different asGC subunits exhibit unique kinetic properties, and their combinations into heterodimers lead to distinct functions (Morton et al. 2005; Morton et al. 2008) . A plausible explanation for our observations is that by inducing the expression of only one subunit, we may be shifting the natural equilibrium of dimers toward one function over the others; however, this remains to be experimentally demonstrated. To investigate whether the effect of Gyc89Db on OL volume was due to altered cell proliferation, we examined the consequences of asGC subunit loss and targeted gain-of-function on NE cell number and proliferation.

Our staining techniques included nuclear staining with methyl green to quantify total cell numbers ( Fig. 1A ), F-actin staining for anatomical segmentation of the NE ( Fig. 1B ), and anti-PH3 staining to quantify mitotic cells ( Fig. 1C ). Despite rigorous examination using confocal microscopy of brains displaying the three markers with outstanding quality (Fig.1 A-C), we found no statistically significant differences in cell number or proliferation when control samples were compared with either the contextual double loss-of-function in *Gyc89Da* and *Gyc89Db* ( *Gyc89Da ^-/-^ Db ^-/-^* ) or the ectopic expression of *Gyc89Db* in neuroepithelial cells under the GAL4 ^c855a^ driver ( Fig. 1E ) compared to *wt* or *w ^1118^* .

These negative results suggest that the previously observed increase in neuroblast numbers and OL volume caused by *Gyc89Db* overexpression (Prieto et al. 2024) may occur without significant alterations in the third-instar NE. Should accelerated NE proliferation have occurred, it likely took place earlier during larval development, possibly alongside compensating waves of neuroblast differentiation that prevented NE from overgrowth.

## Methods

*Fly strains*

Flies were raised on cornmeal medium at 25°C with 12:12 h light:dark cycles, as previously described (Ferreiro et al., 2018) . The wild-type strain ( *wt* ) Vallecas (Izquierdo 1994) and *w ^1118^* (BDSC #5905, Bloomington Drosophila Stock Centre, Bloomington, Indiana, USA) were used as control strains. Expression of *UAS- Gyc89Db* (Vermehren-Schmaedick et al. 2010) in the OL was obtained with the driver *GAL4 ^c855a^* (Egger et al., 2007; BDSC stock #6990 Bloomington *Drosophila* Stock Centre, Bloomington, Indiana, USA). The *UAS- Gyc89Db* as well as the double mutant *Gyc89Da ^-/-^ Db ^-/-^* (Vermehren-Schmaedick et al. 2010) (BDSC #93108) were kind gifts from Prof. David Morton (Oregon Health & Science University, Oregon, USA).

*Immunostaining and image acquisition*

Brains were dissected from wandering third-instar larvae (L3), fixed, and immunostained as previously described (Baccino-Calace et al. 2020) . Primary antibodies to phosphorylated H3 histone (anti-PH3, 1:200, Cell Signaling Technologies #9713) were used as a mitotic marker to assess cell proliferation. Methyl green (Sigma-Aldrich) was used to stain cell nuclei, and rhodamine-conjugated phalloidin (Sigma-Aldrich) was used to stain F-actin as previously described (Prieto et al. 2017) . Fluorescent conjugated secondary antibodies Alexa488, Cy3 and Cy5 from Thermo Fisher were used. Images were acquired with a Zeiss LSM800 Airyscan confocal microscope and processed with FIJI (Schindelin et al. 2012) . Semi-automated OL segmentation was performed with TrakEM2 (Cardona et al. 2012) . Analyses and illustrations were made using R version 4.4.0 on RStudio version 2023.06.01.

## Reagents

Strains: Stocks obtained from the BDSC or kindly provided by colleagues as stated above.

*Gyc89Da ^-/-^ Db ^-/-^* (w[*]; PBac{w[+mC]=RB}Gyc89Da[e01821] Mi{GFP[E.3xP3]=ET1}Gyc89Db[MB03197])

*GAL4 ^c855a^* ( *w[1118]; P{w[+mW.hs]=GawB}C855a* )

*UAS-gyc89Db* ( w[*]; P{w[+mC]=UAS- Gyc89Db .V}2 )
